# Inequalities in Childhood Nutrition, Physical Activity, Sedentary Behaviour and Obesity in Italy

**DOI:** 10.3390/nu15183893

**Published:** 2023-09-07

**Authors:** Angela Spinelli, Laura Censi, Donatella Mandolini, Silvia Ciardullo, Michele Antonio Salvatore, Gianfranco Mazzarella, Paola Nardone

**Affiliations:** 1 National Centre for Disease Prevention and Health Promotion, Italian National Institute of Health (Istituto Superiore di Sanità), Viale Regina Elena 299, 00161 Rome, Italy; donatella.mandolini@iss.it (D.M.); silvia.ciardullo@iss.it (S.C.); micheleantonio.salvatore@iss.it (M.A.S.); paola.nardone@iss.it (P.N.); 2 Council for Agricultural Research and Economics-Research Centre for Food and Nutrition, Via Ardeatina 546, 00178 Rome, Italy; laura.censi@crea.gov.it; 3 ASL Napoli 3 Sud, Via Marconi 66, 80059 Torre del Greco, Italy; gianfranco.mazzarella@regione.campania.it

**Keywords:** fruit and vegetables, sugar-sweetened beverages, physical activity, screen time, overweight, obesity, parental education, residence, citizenship, socio-economic characteristics

## Abstract

Unhealthy diets, physical inactivity and high body mass index (BMI) are preventable risk factors for non-communicable diseases throughout a person’s lifespan. The higher prevalence of these risk factors in children from lower socio-economic groups has been generally observed. The aim of this study is to explore the effect of parents’ socio-economic conditions on children’s consumption of fruit, vegetables and sugar-sweetened drinks, and inactivity, sedentary behaviour, overweight and obesity. This study used data from the sixth cross-sectional survey of the surveillance “OKkio alla Salute” (Italian COSI), involving 2467 schools and 53,275 children in 2019. All the information was collected through four questionnaires addressed to parents, children, teachers and head teachers. The weights and heights of the children were measured with standard techniques and equipment to classify overweight/obesity according to the WOF-IOTF cut-offs. The results showed a high percentage of children who do not adhere to health recommendations and a high prevalence of overweight and obesity. In particular, “less healthy” behaviours and higher BMI were more frequent in children from families with a lower socio-economic status and those residing in Southern Italy. These findings highlight the need for effective interventions that address the differences in these health-related behaviours.

## 1. Introduction

Unhealthy diets, physical inactivity and high BMI are well-recognized harmful and preventable risk factors for non-communicable diseases [1] and it is important to start avoiding them from early life. In fact, they can have direct effects on children’s health, and habits that develop early in life may influence the health and choices that people make later as adults [2,3]. For example, poor nutrition in childhood and adolescence, when bones develop, could influence their growth and put them at risk for bone fractures in later life, regardless of attempts to slow bone loss in adulthood [4].

The consumption of sufficient vegetables and fruits is important for the child’s development and may reduce the risk of cardiovascular diseases, stomach cancer and colorectal cancer in later life [5,6]. Frequent consumption of sugar-sweetened beverages has been linked to harmful health-related factors, such as weight gain, dental caries and insulin resistance in children [7,8].

Physical inactivity is a key behavioural risk factor for cardiometabolic and cardiorespiratory health in children and plays a role in their musculoskeletal fitness, bone development, cognitive outcomes and psychological well-being [9]. Sedentary behaviours in youth, especially screen time, have also received great attention as potential independent risk factors for health outcomes [10].

Moreover, dietary factors, physical inactivity and increased sedentary lifestyle, which often co-occur, contribute to the development of childhood obesity [11]. In the last 30 years, many studies have been conducted on the prevalence of childhood overweight and obesity which have shown, after a great increase, a plateau in many high-income countries, albeit at high levels, but have continued to increase in some sub-groups and other parts of the world [12,13]. The effects of high BMI on physical and psychological health are well documented. Obesity can lead to severe health conditions, including non-insulin-dependent diabetes, cardiovascular problems, bronchial asthma, obstructive sleep apnoea, hypertension, hepatic steatosis, gastroesophageal reflux and psychosocial problems [14]. Moreover, obese children and adolescents are more likely to be obese in adulthood [15].

Finally, contemporary exposure to different unhealthy conditions in childhood and over the course of a lifetime may increase the negative effects on health [16]. Several studies have reported differences in childhood nutrition, physical activity, overweight and obesity in relation to socio-economic status [17,18,19,20,21]. In fact, everything around how children grow and develop, what their life chances are, and their health and well-being, is intricately and inextricably connected to their family and community environments. Generally, studies of socio-economic status have shown a trend towards a higher prevalence of these risk factors in children of lower socio-economic groups [22].

Various indicators of the socio-economic position of parents are used in health research, which are measured at the individual level: education, income, wealth, employment, occupation and composite indicators (e.g., socio-economic status—SES, Family Affluence Scale—FAS). Geographic setting and citizenship can also play an important role in shaping health [23].

The current study aims to explore the effect of parents’ education, citizenship, wealth and geographic area of residence on children’s consumption of fruit, vegetables and sugar-sweetened drinks, physical activity, sedentary behaviour, overweight and obesity in Italy. The reason for analysing these inequalities in Italy is that great differences in socio-economic conditions in the general population are present, especially among geographic areas. Also, because the sample is large (about 50,000 children) and regionally representative, the significance of the residential area and the differences in socio-economic conditions on unhealthy behaviours in childhood can be assessed.

## 2. Materials and Methods

### 2.1. Data

Data from the Italian National Surveillance System “OKkio alla SALUTE”, coordinated by the Istituto Superiore di Sanità (Italian National Institute of Health (INIH)), were used [24]. This cross-sectional survey is part of the Childhood Obesity Surveillance Initiative (COSI) of the World Health Organization (WHO) Regional Office for Europe [25] and its objective is to obtain information on the prevalence of overweight and obesity (by routine measurement of body weight and height) and lifestyle behaviours among primary-school children. All 21 Italian regions participated with representative samples at the regional level or at Local Health Unit (LHU) level. The target population was children in the third grade of primary schools, aged mainly 8 or 9 years. With the support of the Ministry of Education, lists of public and private schools and classes with the number of children in each class were obtained from regional school authorities. A stratified cluster sample design was used, following the WHO cluster survey methodology [26], with classes set as the sampling unit. All children of the selected classes were invited to participate. The sample size was estimated in such a way as to achieve a precision of 3% in the estimate of BMI (the most important outcome variable) for regional estimates and of 5% for LHU estimates. The finite population correction was used because the sample size was large relative to the school children population size, particularly when LHU estimates were made. The sample size estimate was also adjusted by assuming a design effect based on the results of the previous round. The value of the design effect for the BMI was variable among LHUs or regions but almost always its value was no higher than 2.

The Ethics Committee of the INIH approved the protocol and a signed consent from at least one of the parents was required for the child’s participation.

The data collection was conducted by staff of the LHUs. Regional coordinators and LHU staff attended a series of planning meetings and were trained in survey methods in a 2-day training course that included hands-on experience in weighing and measuring techniques, and were provided with guidelines to use during the survey. According to the COSI protocol [27], children were weighed and measured with standard equipment (Seca 872TM scales and Seca 214 stadiometers. Seca GmbH & Co., Hamburg, Germany) and using the same methods. Information on diet, physical activity and sedentary behaviours of children was collected through four questionnaires, one for parents and distributed with the introductory letter and consent form, another for children (distributed with the help of teachers), and one for the teachers and the last for the head teachers. The exclusion criteria were absence from school on the day of the survey and lack of consent of the parents for the children to participate. The data collection occurred between March and June 2019. Further details of the methodology are provided elsewhere [28,29].

### 2.2. Outcomes

A selection of the investigated behaviours was included as outcome variables in this study as well as overweight and obesity. Children were classified as overweight (defined as including obesity) or obese on the basis of their calculated Body Mass Index (BMI), the measured weight in kilograms divided by the height in metres squared, according to the age- and sex-specific WOF-IOTF cut-offs [30]. Among the dietary behaviours, the consumption of (1) fruit and/or vegetables and (2) sugary drinks were selected. Parents were asked how often children consumed them in a typical week, and the possible answers were never, less than once a week, a few days a week (1–3 days), almost every day of the week (4–6 days), once per day, two or more per day (separating 2–3 and 4 or more for fruits and vegetables). In this analysis, the two variables were categorized as “at least once a day” versus “less than once a day”. For physical activity, if either the child or the teacher reported that, the day before the survey, the child had played outside and/or participated in physical activity/sport, the child was classified as “active”. For sedentary behaviours, time spent by the child watching TV and/or playing video games, and playing on a computer, tablet and mobile phone on an ordinary school day (information obtained from the parents’ questionnaire and categorized as ≤2 h and >2 h, as internationally recommended [9]) was used.

### 2.3. Covariates

The socio-economic characteristics included in the analysis were [21,22,31] sex (male or female), area of residence (north, centre, or south), parents’ citizenship (both Italians, one foreign parent, or both foreigners), parents’ educational level (corresponding to the highest level between the two parents and classified as follows: low = less than high school, medium = high school, or high = university degree or more) and economic difficulties (how the family makes ends meet with its earnings: very easily, quite easily, with some difficulty, or with many difficulties).

### 2.4. Statistical Analysis

The frequency distributions of children by socio-economic characteristics and prevalence of the outcome variables stratified by these characteristics were calculated. Final population estimates were weighted to take into account the population of each local health unit or region. Where data were missing, percentages were calculated based on cases with known information. The prevalence by socio-economic characteristics was graphically represented using Kiviat diagrams (or radar plots) [32], and differences were tested by the Pearson design-based χ^2^. Logistic regression models for complex surveys explored the factors associated with each of the outcome variables through the estimation of mutually adjusted odds ratios (ORs) and 95% confidence intervals (CIs). All the analyses were performed using STATA/MP version 15.

## 3. Results

The total sample included 53,275 children enrolled in the 2467 selected schools. Two percent of schools refused to participate, 2738 parents (5.1%) did not allow their children to participate and 3318 children (6.2%) were absent the day of the survey. The data were analysed for the 46,173 children aged 8 or 9 who were at school on the day of the interview and completed their questionnaire.

Table 1 shows the main characteristics of these children. The male to female ratio was approximately 1:1 (51.1% boys vs. 48.9% girls). The majority of children (64.8%) were aged 8 years old and 83.4% had two Italian parents, followed by children who had two foreign parents (10.7%). By design, the regional residence of the sample was the same as the population of Italy: almost half were residents in the northern regions (47.3%), a third in the southern regions (30.8%) and the remaining in Central Italy (21.9%).

More than one third of children had at least one parent with a high level of education, almost half at least one parent with a medium level of education and 16.8% had two parents with a low educational level. About 40% of children lived in families who declared that they have economic problems.

In Table 2, the outcome variables are reported by sex. About a quarter of the sample ate fruit/vegetables less than once a day (boys: 26.0% vs. girls: 22.2%; *p* < 0.001). The proportion of boys who consumed sugary drinks at least once a day was 27.6% compared with 22.2% for girls (*p* < 0.001). A fifth of the children were not physically active the day before the survey and 43.8% of the children (male: 48.5% vs. female: 38.9%; *p* < 0.001) spent more than 2 h a day watching TV or using videogames/tablets/computers/cell phones. The prevalence of overweight (including obesity) was about 30% for both males and for females, but obesity prevalence was higher among boys than girls (9.9% vs. 8.8%; *p* < 0.001).

**Figure 1:**
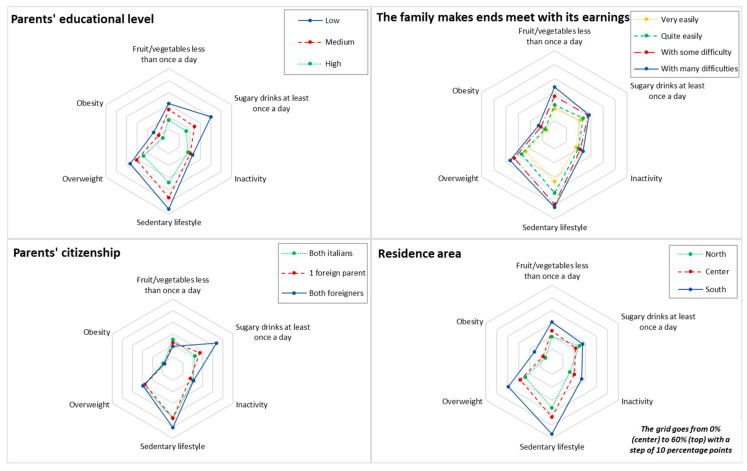
Prevalence of outcome variables by socio-economic characteristics. Italy, 2019.

The prevalence of outcome variables by socio-economic characteristics are reported in Figure 1 and in Table S1 in the Supplementary Materials. In general, the proportion of children with “less healthy” behaviours was higher among children with lower parental education and lower perceived family wealth.

The prevalence of overweight (including obesity) decreased from 36.7% for children whose parents were less educated to 23.9% for children with highly educated parents; for obesity, the prevalence varied from 14.6% to 5.6%, respectively. Children with less educated parents also ate less fruit and vegetables daily (low: 31.1%, medium: 26.2%, high: 17.5%; *p* < 0.001), and consumed more sugary drinks daily (low: 40.4%, medium: 24.6%, high: 16.7%; *p* < 0.001). These children tended to be less active and more inclined to sedentary behaviours. A similar pattern was observed by stratifying for perceived wealth with worse values among families with low perceived wealth, although the differences were less evident.

There were no differences in childhood inactivity, overweight and obesity in terms of parents’ citizenship, while children with both foreign parents consumed more fruit/vegetables and sugary drinks daily than children with two Italian parents. Furthermore, the prevalence of children watching TV or using videogames/tablets/computers/cell phones for more than 2 h on a normal school day was higher among children with two foreign parents in comparison to children with two Italian parents (50.8% vs. 42.9%).

Children who lived in northern regions had healthier habits than those living in central or southern regions, particularly for sedentary lifestyles (56.8% in the South vs. 36.0% in the North). The prevalence of obesity was also higher in the south (15.7%) compared to the centre (8.0%) and the north (5.9%).

The results of the logistic regression models (Table 3) were similar to most of the unadjusted results. The majority of unhealthy habits were more prevalent among male children (fruit/vegetables less than once a day, OR_adj_: 1.26, 95%CI 1.18–1.34; sugary drinks at least once a day, OR_adj_: 1.38, 95%CI 1.29–1.47; sedentary lifestyle, OR_adj_: 1.54, 95%CI 1.46–1.62). Parent’s educational level and area of residence showed the biggest differences. For example, the prevalence of obesity was more than double among children whose parents were less educated in comparison to children with highly educated parents (OR_adj_: 2.33, 95%CI 2.03–2.66) and among children residing in the south vs. those resident in the north (OR_adj_: 2.80, 95%CI 2.52–3.11), after adjusting for all the other variables in the model. Children with parents with a low education, those residing in the south and children with families in economic difficulties had a higher risk of low consumption of fruit and vegetables. On the contrary, this risk was lower among children with two foreign parents in comparison to those with two Italian parents (OR_adj_: 0.73, 95%CI 0.65–0.81). The risk of consumption of sugary drinks at least once a day was very high among children with parents with a low education (OR_adj_: 2.95 95%CI 2.68–3.24) and with two foreign parents (OR_adj_: 2.50 95%CI 2.26–2.76). For physical inactivity, the variable which showed an important increase in the risk was the area of residence (OR_adj_: 1.94 in the south and OR_adj_: 1.32 in the centre). The risk of sedentary behaviour increased with decreasing parental education, in the residents in the south, among children with two foreign parents, and with families with economic difficulties.

## 4. Discussion

This study, based on a nationally representative sample of almost 50,000 children, shows that many children in Italy do not follow the recommendations on nutrition, physical activity and sedentary behaviour and their prevalence of overweight and obesity are high. The results also show that the “less healthy” behaviours were more frequent in children in families with less parental education and less perceived family wealth and, for consumption of sugary drinks and sedentary behaviour, where both parents are foreign. Differences in the proportions of children with unhealthy habits depended on the area of residence, with the highest values in children living in Southern Italy, the most deprived part of the country [33]. These findings highlight the need for effective interventions that address the differences in these health-related behaviours.

### 4.1. Nutrition

According to our analyses, about a quarter of the children in Italy in 2019 ate fruit and vegetables less than once a day and consumed sugary drinks at least once a day. The low consumption of fruit and vegetables, with a decreasing trend from northern to southern Italy, is in line with the results of the national survey on aspects of daily life conducted by the Italian National Institute of Statistics (ISTAT) in 2020, where overall, 18.7% of people aged 3 and over did not eat fruit and vegetables daily [34]. Moreover, in 2019, only 5.7% of children [35] consumed at least five portions of fruit/vegetables (≥400 g) per day, as recommended by WHO [36]. Our results on the consumption of sugary drinks are not comparable with those of ISTAT, which was based on children aged over 10; however, in the 11–13 age group, 23.4% consumed carbonated drinks daily in 2016–2017, close to the value of sugary drinks in children aged 8–9 years found in our study. The data from the Health Behaviour in School-aged Children (HBSC) collected in 2018 in Italy showed a lower prevalence among adolescents: 15.9% of boys and 11.3% of girls aged 11–15 drank carbonated sugary beverages at least once a day [37].

Compared to the results of the other countries which participated in the WHO COSI in 2018–2020, children living in Italy were within the average values for fruit consumption at least once a day, with a slightly lower vegetable consumption [25]. The percentage of children in Italy who consumed sugary drinks more than three days a week was lower than the average value in all countries [25]. Our analyses have shown a greater consumption in Southern Italy.

As in our study, several authors have found an association between unhealthy eating habits regarding the consumption of fruit, vegetables and sugary drinks, and low socio-economic status, with differences between countries and geographic areas [17,18,31,38]. The Feel4 Diabetes study of about 12,000 European children showed that socio-economic vulnerability is related to an increased risk of unhealthy eating habits in children [17]. Similarly, the ISTAT survey found that, as the educational qualification of the parents increases, the consumption of fruit, vegetables and drinks improve in children [34]. The IDEFICS study found a similar association [39]. A positive association between maternal education and adherence to Mediterranean diet, mainly characterized by plant-based food, was observed in 8–9-year-old children residing in Italy, especially in Southern Italy, by Roccaldo et al. [40].

A review of studies of dietary habits and determinants of adherence to a Mediterranean diet in southern European children found a general trend to move away from this traditional food model over time, towards unhealthy food choices, likely due to the socio-economic changes in recent years [41].

Our findings about food habits in children with foreign parents are in line with those by Archero et al. [42], who found a mixed eating behaviour in children and adolescents of other ethnic origins living in Northern Italy. They tended to maintain a higher intake of more traditional foods, such as fish, cereals or grain for breakfast, and yoghurts and/or cheese, suggesting more home-prepared foods in their family environment. On the other hand, they frequently ate at fast-food restaurants, skipped breakfast, consumed commercially baked goods for breakfast, or sweets and candy several times per day.

Indeed, dietary habits are strongly influenced by multiple interacting factors related to children’s living environment, including socio-economic and cultural factors, time constraints, ethnicity, food availability, portion sizes and meal context (e.g., watching TV during meals), and parents play a direct role through their behaviours, attitudes, eating styles [43,44] and satisfaction with body image [3]. As children’s eating habits are shaped early and may persist into later ages, prevention programs should be addressed to parents, taking into account different socio-economic and educational levels [45].

### 4.2. Physical Activity and Sedentarity

In our study, one child out of five was not physically active the day before the survey and almost half of the children spent more than 2 h engaged in sedentary activity. Our results are similar to those obtained by ISTAT in 2017–2018, where 18.2% of boys and 20.6% of girls aged 6–10 years did not practice any sport or physical activity [46]. These low levels of physical activity were confirmed among adolescents: only 12.2% of boys and 6.8% of girls aged 11–15 years declared that they engaged in moderate-to-vigorous physical activity every day in Italy in 2018 [47]. Because of the different definitions, it is not possible to compare our indicator of inactivity with those of the other 28 countries participating in COSI in 2018–2020 [25]. However, the percentage of children practicing sports/dancing for at least 2 h a week in Italy in 2019 was higher than the average found in COSI (70% vs. 53%), but the proportion of children travelling actively to and from school was lower (25% vs. 41%).

Our study also shows that 43.8% of children spent more than two hours a day watching television or using electronic devices on schooldays (or weekdays). Compared with other countries participating in COSI, the proportion of children spending at least two hours a day on weekdays and weekends watching television or using electronic devices in Italy was among the highest (72% vs. 43%).

Our analyses show a strong geographic trend with increasing occurrence of inactivity and sedentary lifestyle from north to south Italy. We have also found an association with parents’ education, citizenship and wealth, especially for the screen time, as in other studies. Musić Milanović et al. [19] found that in the WHO European Region, low socio-economic status children were less likely to participate in sports clubs and more likely to have more than 2 h/day of screen time. A study where physical activity was measured using accelerometers revealed significantly higher levels of childhood vigorous physical activity accumulated as maternal education and annual household income increased [48]. Additionally, children from certain minority ethnic groups accrued less daily physical activity compared with their British counterparts. In a prospective cohort study in the Netherlands, children of mothers with a low education or low-income households were more likely to be in the “high screen time and physically inactive” cluster [49]. These differences can partly be due to the environment where they live, without play parks, walkable neighbourhoods, cycle lanes, low traffic areas and schools without a gym or playground, all discouraging physical activity. For example, the results of OKkio alla SALUTE showed that 36.7% of the schools did not have a gym and 36.4% did not have a playground in Southern Italy, while these percentages were 19.9% and 16.4% in Northern Italy in 2019 [35]. The family styles can also have an impact on children’s physical activity and sedentarity, with children of parents who are less physically active and more sedentary tending to follow their parents’ behaviour [50].

### 4.3. Overweight and Obesity

The prevalence of childhood overweight and obesity in Italy is amongst the highest in Europe [25], although there has been a general slight decrease in the prevalence of childhood overweight and obesity from 2008 to 2019 in Italy [28]. The results of this study show strong differences according to the socio-economic conditions of the child’s family. If parental education and family wealth play a similar role on overweight, for obesity the level of education is more important. Social inequalities in obesity are well known for many rich OECD (Organization for Economic Co-operation and Development) countries [51]. In general, the prevalence of childhood obesity in Europe is higher in lower socio-economic families [20,52] and the gap between groups seems to increase over time [52,53,54].

Another important factor for childhood overweight and obesity in Italy is the area of residence, with children living in the south at almost three times the level of those living in the north, and double that of residents in the central regions, regardless of the other socio-economic conditions of the families. This can be explained not only by different eating habits and lack of physical activity (as shown by the other results of this study), but also by factors of a cultural nature and the higher prevalence of excess weight among parents. A recent study has shown that the prevalence of adult overweight and obesity is higher in the southern regions [55] and all rounds of OKkio alla SALUTE have found that overweight and obese children tend to have overweight and obese parents [30]. Living in a society where overweight and obesity are very common can also negatively influence the prevalence of excess of weight among children and the perception of the problem among their parents [56]. Immigration from various low-income countries began in Italy in the 1990s. In 2022, the larger foreign communities were Romanians, Moroccans, Albanians and Chinese. These countries have very different cultures and nutritional habits, but in this study, the parents’ citizenship, after adjusting for the other socio-economic conditions, did not show a significant effect on childhood overweight and obesity, in contrast to other countries where ethnic differences have been observed [57,58].

### 4.4. Limitations and Strengths of the Study

One limitation of the study is the inclusion of only third-grade primary school children in Italy, aged 8–9 years, and the results are not necessarily generalizable to other age groups or populations. Some of the variables were self-reported or reported by teachers or parents, and these might not have been completely objective. The use of BMI as the sole indicator of overweight and obesity might be a limitation because it does not differentiate between body fat and muscle mass. However, this is critical in clinical evaluation where a single individual is investigated, but less important in epidemiological studies of association and monitoring. The survey was conducted immediately before the COVID-19 pandemic and the economic consequences of the Ukraine conflict which may have subsequent effects on these results.

This study has also some strengths. First of all, the large sample was representative at the regional level which allows for the evaluation of the importance of area of residence and the differences due to socio-economic conditions. The high response rate of schools and parents and the low number of missing data points in each category of the variables limited the selection biases. The percentage of absent children on the day of the survey was similar to that of a normal day. Finally, the children were weighed and measured with standard equipment and using the same methods by trained personnel.

## 5. Conclusions

Overall, our findings highlight the importance of area of residence and socio-economic conditions for childhood nutrition, physical activity, sedentary behaviour and obesity. There are several primary causal pathways through which these factors can affect unhealthy habits: shared environments (built, social and economic) and norms, attitudes, beliefs and behaviours of people living in the same place or with a similar background. One important way to intervene is through support in the early years, improving education and parenting (e.g., antenatal care, breastfeeding, early reading and good quality preschools). School is a setting where inequalities can be contrasted, with the promotion of healthy eating and active lifestyles through children’s and parents’ education. With school small rewards, children could be encouraged to taste fruit and vegetables repeatedly, giving them the opportunity to develop a liking for these foods. At least half an hour a day of physical activity should be mandatory in all primary schools. Finally, the social environment should be improved, especially in the south and in more deprived areas, making healthy food more available and building playgrounds, gyms, cycling roads and other infrastructure which encourage physical activity.

## Figures and Tables

**Table 1:** Sample characteristics. Italy, 2019.

Variables	N = 46,173 %
**Child’s sex**	
Male	51.1
Female	48.9
*Missing*	*0.7*
**Child’s age**	
8 years	64.8
9 years	35.2
**Parents’ educational level**	
Low	16.8
Medium	48.5
High	34.7
*Missing*	*11.1*
**Area of residence**	
North	47.3
Centre	21.9
South	30.8
**Parents’ citizenship**	
Both Italians	83.4
One foreign parent	5.8
Both foreigners	10.7
*Missing*	*7.5*
**The family makes ends meet with its earnings**	
Very easily	12.2
Quite easily	46.2
With some difficulty	34.9
With many difficulties	6.7
*Missing*	*6.6*

**Table 2:** Outcome variables by sex. Italy, 2019.

Outcome	Males N = 23,417 %	Females N = 22,474 %	Total N = 46,173 * %	*p*-Value
**Fruit/vegetables**				
Less than once a day	26.0	22.2	24.1	<0.001
At least once a day	74.0	77.8	75.9	
*Missing*	*4.3*	*3.8*	*4.1*	
**Sugary drinks**				
At least once a day	27.6	22.2	25.0	<0.001
Less than once a day	72.4	77.8	75.0	
*Missing*	*6.2*	*5.6*	*5.9*	
**Inactivity ^(1)^**				
Yes	19.7	20.9	20.3	0.015
No	80.3	79.1	79.7	
*Missing*	*1.6*	*1.7*	*1.7*	
**Sedentary lifestyle ^(2)^**				
Yes	48.5	38.9	43.8	<0.001
No	51.5	61.1	56.2	
*Missing*	*11.3*	*11.4*	*11.3*	
**Overweight ^(3)^**				
Yes	29.9	29.7	29.8	0.741
No	70.1	70.3	70.2	
*Missing*	*0.4*	*0.3*	*1.0*	
**Obesity**				
Yes	9.9	8.8	9.4	<0.001
No	90.1	91.2	90.6	
*Missing*	*0.4*	*0.3*	*1.0*	

* A total of 282 cases with unknown information on sex. ^(1)^ Physical inactivity (not playing outdoors and not doing physical activity) on the day before the interview. ^(2)^ Watching TV or using videogames/tablet/computers/cell phones for more than 2 h on a normal school day. ^(3)^ Overweight including obesity.

**Table 3:** Mutually adjusted * odds ratios for the reported variables—logistic regression models. Italy, 2019.

Variables	Fruit/Vegetables Less Than Once a Day		Sugary Drinks at Least Once a Day		Inactivity ^(1)^		Sedentary Lifestyle ^(2)^		Overweight ^(3)^		Obesity	
	Yes vs. No		Yes vs. No		Yes vs. No		Yes vs. No		Yes vs. No		Yes vs. No	
	OR_adj_	95%CI	OR_adj_	95%CI	OR_adj_	95%CI	OR_adj_	95%CI	OR_adj_	95%CI	OR_adj_	95%CI
**Child’s sex**												
Female	1		1		1		1		1		1	
Male	1.26	1.18–1.34	1.38	1.29–1.47	0.94	0.88–1.00	1.54	1.46–1.62	1.03	0.97–1.09	1.16	1.07–1.26
**Parents’ educational level**												
High	1		1		1		1		1		1	
Medium	1.58	1.46–1.70	1.57	1.45–1.70	1.11	1.02–1.19	1.56	1.46–1.66	1.31	1.23–1.40	1.65	1.47–1.85
Low	1.93	1.76–2.13	2.95	2.68–3.24	1.16	1.04–1.29	2.04	1.87–2.23	1.58	1.45–1.71	2.33	2.03–2.66
**Area of residence**												
North	1		1		1		1		1		1	
Centre	1.33	1.23–1.45	0.88	0.81–0.97	1.32	1.13–1.54	1.41	1.3–1.52	1.27	1.18–1.37	1.38	1.21–1.56
South	1.73	1.62–1.86	1.28	1.19–1.39	1.94	1.69–2.23	2.41	2.24–2.59	1.99	1.86–2.13	2.80	2.52–3.11
**Parents’ citizenship**												
Both Italians	1		1		1		1		1		1	
One foreign parent	0.90	0.79–1.03	1.39	1.23–1.57	0.90	0.78–1.04	1.06	0.95–1.19	0.99	0.88–1.11	1.05	0.87–1.27
Both foreigners	0.73	0.65–0.81	2.50	2.26–2.76	1.16	1.02–1.33	1.54	1.39–1.71	1.07	0.98–1.18	1.07	0.92–1.24
**The family makes ends meet with its earnings**												
Very easily	1		1		1		1		1		1	
Quite easily	1.09	0.99–1.20	1.03	0.93–1.15	1.05	0.94–1.17	1.28	1.16–1.41	1.10	1.00–1.21	1.07	0.91–1.25
With some difficulty	1.36	1.23–1.51	1.04	0.93–1.17	1.08	0.96–1.21	1.50	1.36–1.66	1.31	1.20–1.44	1.35	1.15–1.58
With many difficulties	1.82	1.58–2.10	1.02	0.89–1.18	1.32	1.13–1.53	1.58	1.38–1.82	1.54	1.34–1.76	1.60	1.29–1.97

* Odds ratios adjusted for all variables in the first column of the table. ^(1)^ Physical inactivity (not playing outdoors and not doing physical activity) on the day before the interview. ^(2)^ Watching TV or using videogames/tablets/computers/cell phones for more than 2 h on a normal school day. ^(3)^ Overweight including obesity.

## Data Availability

OKkio alla SALUTE data and questionnaires can be accessed via a request to the Principal Investigator, Dr. Paola Nardone: paola.nardone@iss.it. For further information, please see https://www.epicentro.iss.it/okkioallasalute/ (accessed on 4 July 2023).

## Supplementary Materials

The following supporting information can be downloaded at: https://www.mdpi.com/article/10.3390/nu15183893/s1, Table S1: Prevalence of outcome variables by socio-economic characteristics. Italy, 2019.
