# Supplementary material for: Inequalities in Childhood Nutrition, Physical Activity, Sedentary Behaviour and Obesity in Italy
Source: Nutrients. 2023 Sep 7;15(18):3893. doi: 10.3390/nu15183893 (PMC10534384; doi:10.3390/nu15183893)
Supplement: Supplementary file 1 [file nutrients-15-03893-s001.zip › nutrients-2573928-supplementary.pdf]

Table S1. Prevalence of outcome variables by socio-economic characteristics. Italy, 2019

Socio-economic characteristics	Fruit/vegetables less than once a day		Sugary drinks at least once a day		Inactivity (1)		Sedentary lifestyle (2)		Overweight (3)		Obesity	
	%	p-value	%	p-value	%	p-value	%	p-value	%	p-value	%	p-value
**Parents' educational level**												
Low	31.1	<0.001	40.4	<0.001	22.7	<0.001	55.9	<0.001	36.7	<0.001	14.6	<0.001
Medium	26.2		24.6		20.5		46.5		30.7		9.6	
High	17.5		16.7		18.3		33.9		23.9		5.6	
**Parents' citizenship**												
Both italians	24.9	<0.001	22.0	<0.001	20.3	0.045	42.9	<0.001	29.6	0.188	9.3	0.205
1 foreign parent	22.2		26.9		17.3		42.6		27.5		8.3	
Both foreigners	19.0		43.6		20.7		50.8		29.3		8.5	
**The family makes ends meet with its earnings**												
Very easily	18.5	<0.001	21.2	<0.001	17.7	<0.001	33.3	<0.001	23.8	<0.001	6.5	<0.001
Quite easily	21.4		23.5		19.6		41.6		27.4		7.8	
With some difficulty	27.7		27.6		21.1		49.7		33.4		11.4	
With many difficulties	34.0		28.3		23.6		51.7		36.6		13.4	
**Area of residence**												
North	19.4	<0.001	24.7	<0.001	16.0	<0.001	36.0	<0.001	24.1	<0.001	5.9	<0.001
Center	24.2		21.4		20.1		43.3		28.6		8.0	
South	31.2		27.9		27.0		56.8		39.4		15.7	

(1) Physical inactivity (not playing outdoors and not doing physical activity) in the day before the interview, (2) Watching TV or using videogames/tablet/computers/cellphones for more than 2 hours on a normal school day, (3) Overweight including obesity
